# The burden of cancer in Latin America and the Caribbean: Time for planning a better cancer control

**DOI:** 10.1016/j.lana.2022.100336

**Published:** 2022-07-29

**Authors:** Alejandro Mohar-Betancourt

**Affiliations:** a Unidad de Investigación Biomédica en Cáncer, Instituto Nacional de Cancerología, México; b Instituto de Investigaciones Biomédicas, Universidad Nacional Autónoma de México, México

Based on information published in Globocan, in the year 2020 more than 18 million new cases of cancer were registered worldwide, and 8 million deaths were associated with neoplastic disease.^1^

In *The Lancet Regional Health - Americas*, Piñeros et al.^2^ use this information to calculate the incidence and mortality rates in the region of Latin America and the Caribbean, where a population of 685 million is distributed in 32 countries, and life expectancy varies from 65 to 83 years. In this region, cancer is among the first causes of death. Analysis of the death tendencies in this group of countries allows for an evaluation of the prevention efforts, early diagnosis and treatment for the thousands of patients diagnosed with cancer every year. It is a priority to determine the epidemiology of these diseases to establish public health policies and actions that can contribute to better control of cancer in this region.

Doubtlessly, the region of Latin America and the Caribbean share points in common such as language, miscegenation, economic development, and cultural aspects. At the same time, the region displays significant variations such as in geography, economic resources, the speed of epidemiological transition, and great diversity in the access to treatment and the quality of oncological medical attention.^3^

Piñeros et al.^2^ pinpoint the magnitude of cancer in Latin America and the Caribbean. They present incidence and mortality rates by type of tumour, country, and gender, as well as the evolution of mortality by tumour during the past four decades, and compare them with data from Spain and the United States, both highly developed countries that illustrate the challenges and opportunities for this region. They project the growth of the cancer epidemic in Latin America and the Caribbean to 2040, under the premise that the incidence rates would remain stable. Faced with the absence of population-based cancer records, they direct their efforts to obtain epidemiological data based on incidence and mortality rates. It is imperative to further support the development of Population-Based Cancer Registries in this region.^4^

As in other regions of the world, the most frequent tumours are prostate, breast, colorectal, lung and stomach cancer. These amount to 48% of the 1.5 million patients with a cancer diagnosis in 2020.^2^ In the Latin America and Caribbean region, important differences in the incidence rate were detected; for example, lung cancer varied up to 10-fold in countries such as Cuba, Uruguay and Argentina compared with Guyana.^2^

Worthy of attention is the high mortality rate of malignant tumours associated with infections such as stomach and cervical cancer. The latter showed an upsurge in incidence rates in Argentina, Brazil, and Uruguay, causing the death of 31,582 women in 2020. This finding constitutes an important challenge, considering the commitment established by the WHO Cervical Cancer Elimination Strategy to reducing the incidence of this neoplasia to 4/100,000.^5^

Today we know that space for primary and secondary prevention is within reach. Reduction of smoking by increasing the price of tobacco is a fundamental factor to diminish the incidence of malignant tumours.^6^ Control of cancer-associated infections is forceful such as human papillomavirus, *Helicobacter pylori* –the causal agent of 90% of the cases of stomach cancer–, as well as B and C hepatitis –causal agents of liver cancer.^7^^,^^8^ The two latter tumours cause the death of 90,000 patients each year.^2^

Latin America and the Caribbean have focused efforts to organize screening programs for cervix and mammary cancer detection. Results are not yet favourable due to low coverage, lack of human resources and supplies necessary for diagnostic tests, as well as a slow reference to oncological centres.^3^ If this region does not invest enough economic resources and efficient epidemiological surveillance programs it will not be able to reach cancer control. The profound inequity of access to oncological medical services will continue in these 32 countries, and the 2.4 million cancer patients projected for 2040 by Piñeros et al.^2^ will be inevitable.

Hence, the International Agency of Research on Cancer (IARC) has summoned a group of experts from the region to discuss and generate a **Latin American and Caribbean Code Against Cancer**.^9^ It derives from the European Code Against Cancer,^10^ but analyses the necessities of the region with the aim of reaching maximum benefits in the control of the risk factors for its millions of inhabitants.

Finally, the publication by Piñeros et al.^2^ arrives in a timely manner, coinciding with the concern by IARC about the course that cancer has taken in this region, and the decision to join up with local specialists to reduce the risk of cancer. The results published by Piñeros et al.^2^ are the best instrument and evidence available to develop an optimal plan for the prevention, diagnosis, treatment, and palliative care for benefit of the thousands of patients who struggle daily against cancer.

## Contributors

Alejandro Mohar was the only author of this manuscript, he conceptualized, wrote, edited, reviewed, and approved the final version for submission.

## Declaration of interests

None.
